# Coupled Attitude-Orbit Dynamics and Control for an Electric Sail in a Heliocentric Transfer Mission

**DOI:** 10.1371/journal.pone.0125901

**Published:** 2015-05-07

**Authors:** Mingying Huo, Jun Zhao, Shaobiao Xie, Naiming Qi

**Affiliations:** Department of Aerospace Engineering, Harbin Institute of Technology, Harbin, Heilongjiang, People’s Republic of China; Politehnica University of Bucharest, ROMANIA

## Abstract

The paper discusses the coupled attitude-orbit dynamics and control of an electric-sail-based spacecraft in a heliocentric transfer mission. The mathematical model characterizing the propulsive thrust is first described as a function of the orbital radius and the sail angle. Since the solar wind dynamic pressure acceleration is induced by the sail attitude, the orbital and attitude dynamics of electric sails are coupled, and are discussed together. Based on the coupled equations, the flight control is investigated, wherein the orbital control is studied in an optimal framework via a hybrid optimization method and the attitude controller is designed based on feedback linearization control. To verify the effectiveness of the proposed control strategy, a transfer problem from Earth to Mars is considered. The numerical results show that the proposed strategy can control the coupled system very well, and a small control torque can control both the attitude and orbit. The study in this paper will contribute to the theory study and application of electric sail.

## Introduction

**Fig 1:**
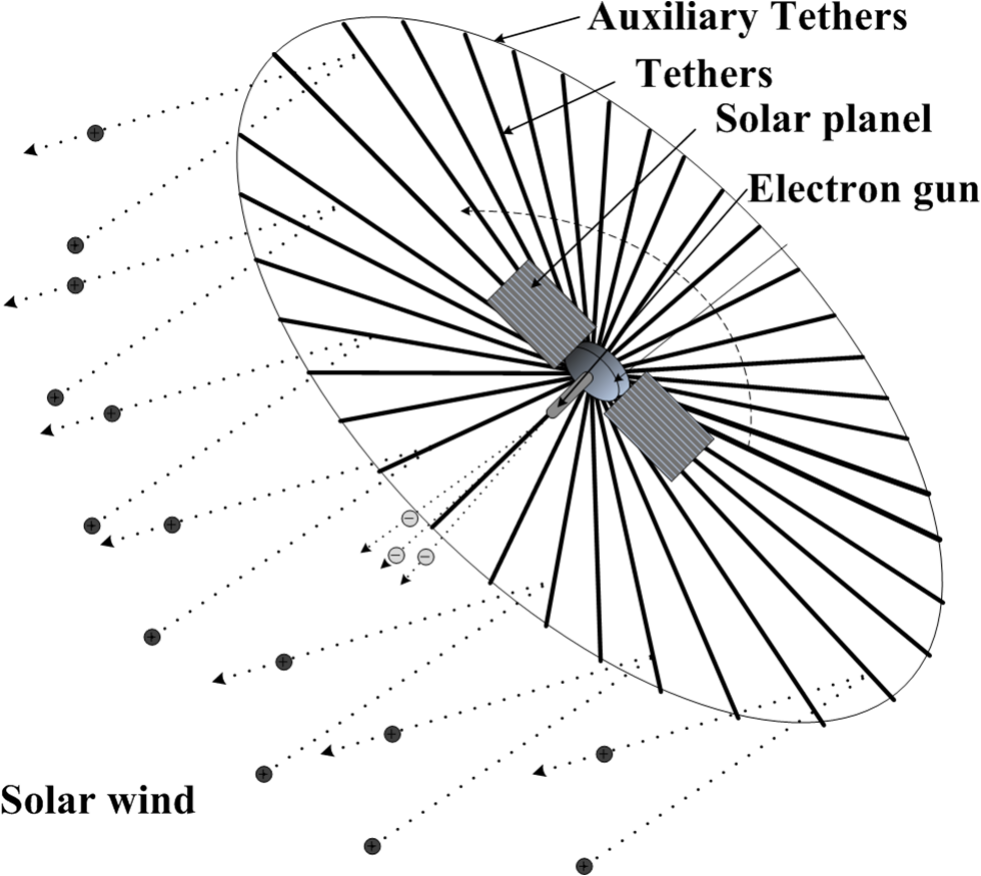
Conceptual sketch of an electric sail.

The electric solar wind sail, electric sail for short, is an innovative propulsion concept. Similar to the more conventional solar sail, it can produce continuous and propellant-less thrust. Unlike the solar sail, the electric sail is accelerated by the solar wind dynamic pressure, instead of the photon momentum. As seen in Fig 1, the electric sail consists of many tethers which are held at a high positive potential through a solar-powered electron gun. The electric field generated by the charged tethers can reflect the solar wind ions to generate thrust without any reaction mass. The deployment and maintenance of these long tethers are implemented by spinning the sailcraft about the symmetry axis. Because electric sails can operate nominally over indefinitely long periods, the achievable energy changes are substantial, and greater than those conventional (either chemical or electrical) propulsion systems [1].

The electric sail was first proposed by Jauhunen in [2] as a propulsion system that uses the solar wind momentum flux for spacecraft thrust, as inspired by the earlier magnetic sail concept [3]. It is noticed that an electric field potential structure with a spatial scale larger than 100m can be created around a thin tether with thickness of a few tens of micrometers. The existing thrust law for a positively charged electric sail tether is based on the studies in [4–6]. Theoretical analysis and experimental researches show that the thrust force of the solar sail decays as (1/*r*)^2^, and that of the electric sail decays as 1/*r*, where *r* is Sun-spacecraft distance. Therefore, for a given characteristic acceleration, an electric sail can provide a greater thrust than a solar sail, if the solar distance is greater than 1 astronomical unit (au). An in-depth analysis of the electric sail performance can be found in [7].

Recently, it was shown that if flight control is accomplished, the navigation in real solar wind conditions to planetary targets with an electric sail is feasible [8]. Therefore, the research on the attitude and orbital control is urgent and necessary. Toivanen [9] discussed the control of a single electric sail tether, and obtained a solution for the voltage modulation that maintains any realistic angle under constant solar wind. However, the attitude control of the whole electric sail and the relationship between the thrust vector and the sail angle were not studied. Like solar sails [10], the orbit and attitude of electric sails should not be completely independent on each other, since the solar-wind-dynamic pressure acceleration induced by the sail attitude will affect the orbit.

The relationship between the thrust vector and the attitude of the electric sail has not been discussed yet, and the orbital and attitude dynamics of the electric sails are investigated separately in most published literatures. Therefore, the coupled attitude-orbit dynamics and control should be investigated. In this paper, the relationship between the thrust vector and the sail attitude is studied first. Based on the thrust model, the coupled attitude-orbit dynamics and control is investigated. Since the above-mentioned research contents haven’t been studied in published literatures and the research on the flight control is urgent in the application of electric sail, the research contents of this paper are necessary and significant. The study in this paper will contribute to the theory study and application of electric sail.

The paper is organized as follows. First of all, the mathematical model characterizing the propulsive thrust as a function of the orbital radius and the sail angle is described. Secondly, since the solar-wind-dynamic pressure acceleration induced by the sail attitude can decide the orbit, the coupled attitude-orbit dynamics of electric sails are discussed. Based on the coupled equations, the flight control is investigated, wherein the orbital control is studied in an optimal framework via a hybrid optimization method and the attitude controller is designed based on feedback linearization control. To verify the effectiveness of the proposed control strategy, transfer problem from Earth to Mars is considered in the paper.

## Thruster Mathematical Model

In previous research, the thrust mathematical model is only characterized by the sun-spacecraft distance [6]. In this section, the research is mainly focus on the corresponding relationship between the thrust vector and the attitude of the electric sail. For simplicity, the flexibility of tethers is ignored in this paper.

### Reference Frame

**Fig 2:**
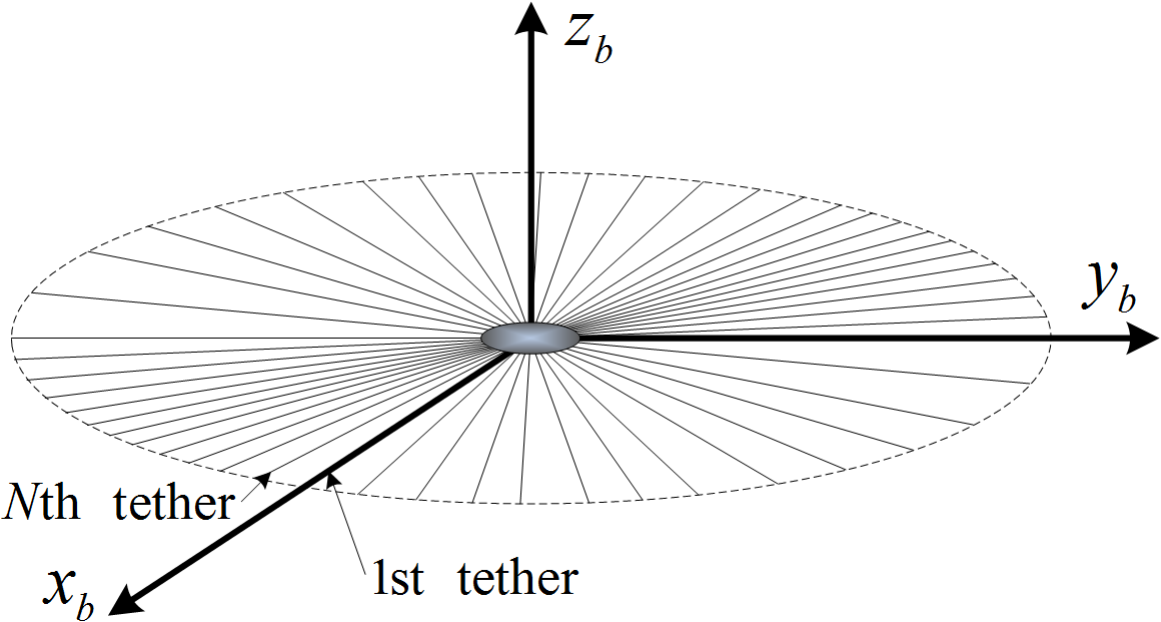
Body reference frame.

**Fig 3:**
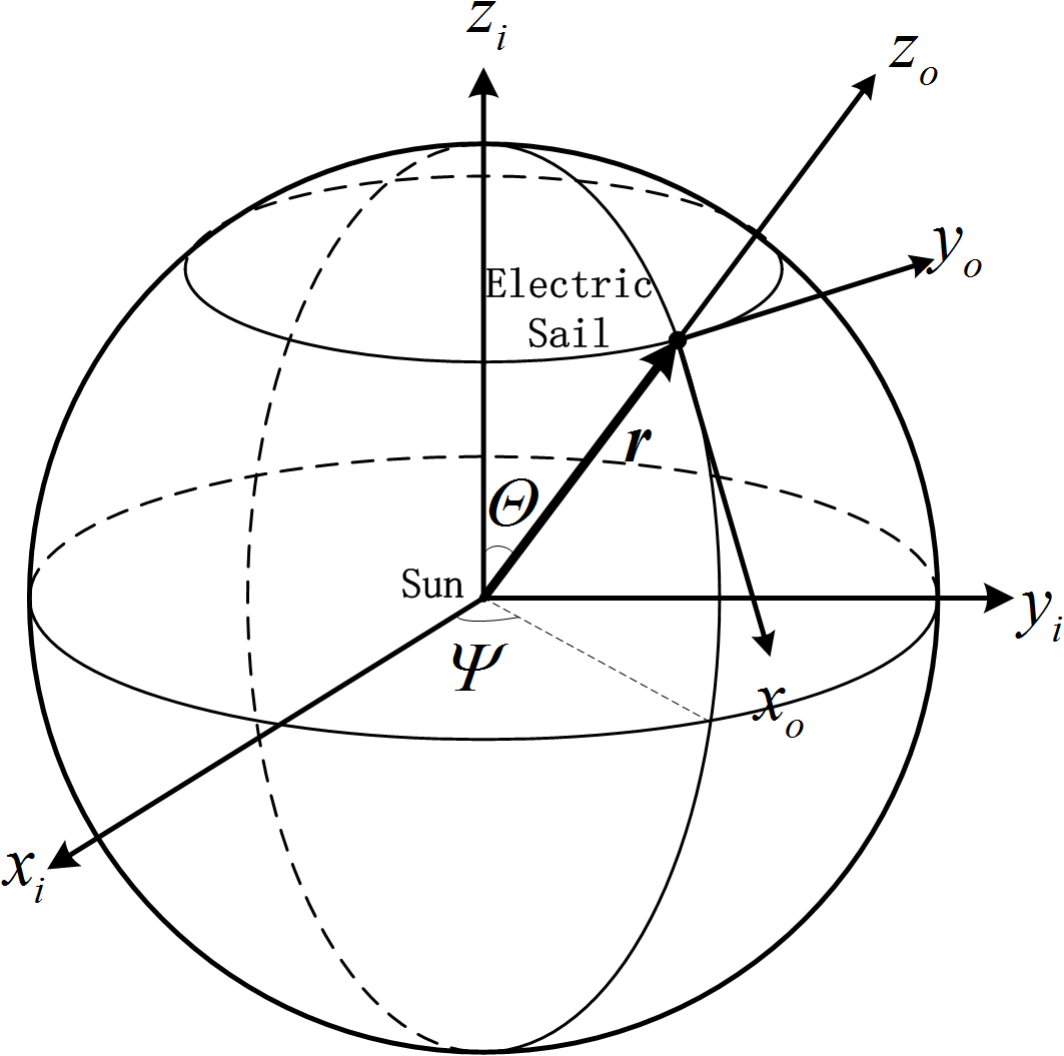
Heliocentric-ecliptic inertial frame and orbital reference frame.

Before the description of the thrust model and the coupled dynamics, three reference frames are introduced, which are the body frame *o*_*b*_ *x*_*b*_ *y*_*b*_ *z*_*b*_, the orbital frame *o*_*o*_ *x*_*o*_ *y*_*o*_ *z*_*o*_ and the heliocentric-ecliptic inertial frame *o*_*i*_ *x*_*i*_ *y*_*i*_ *z*_*i*_. Considering an electric sail consists of *N* tethers as seen in Fig 2, these tethers can be numbered in counterclockwise. The origin of the body frame *o*_*b*_ *x*_*b*_ *y*_*b*_ *z*_*b*_ is at the center-of-mass of the sail, and the *x*_*b*_-axis is in the direction of a given reference tether. The *z*_*b*_-axis is along the normal of the sail, and the *y*_*b*_-axis forms a right-handed system. The orbital frame *o*_*o*_ *x*_*o*_ *y*_*o*_ *z*_*o*_ and the inertial frame *o*_*i*_ *x*_*i*_ *y*_*i*_ *z*_*i*_ are shown in Fig 3. The origin of the orbital frame *o*_*o*_ *x*_*o*_ *y*_*o*_ *z*_*o*_ is at the center-of-mass of the sail and the *z*_*o*_-axis is along the sun-spacecraft direction. The *y*_*o*_-axis is perpendicular to the normal of the ecliptic plane and the *z*_*o*_-axis, and the *x*_*o*_-axis forms a right-handed triad. The origin of the inertial frame *o*_*i*_ *x*_*i*_ *y*_*i*_ *z*_*i*_ is at the center-of-mass of the sun, and the *x*_*i*_-axis is in the direction of sun-equinox. The *z*_*i*_-axis is along the normal of the ecliptic plane, and the *y*_*i*_-axis forms a right-handed system.

The attitude of the sail in the orbital frame can be described by three angles *ϕ*, *θ* and *ψ*, and the rotation sequence from the orbital frame to the body frame is *x*(*ϕ*) → *y*(*θ*) → *z*(*ψ*). Based on the basic knowledge of matrix transformation, the transition matrix from *o*_*o*_ *x*_*o*_ *y*_*o*_ *z*_*o*_ to *o*_*b*_ *x*_*b*_ *y*_*b*_ *z*_*b*_ is

$$A_{bo}(\phi ,\theta ,\psi )=R_{z}(\psi )R_{y}(\theta )R_{x}(\phi )$$ (1.)

### Thrust Vector

**Fig 4:**
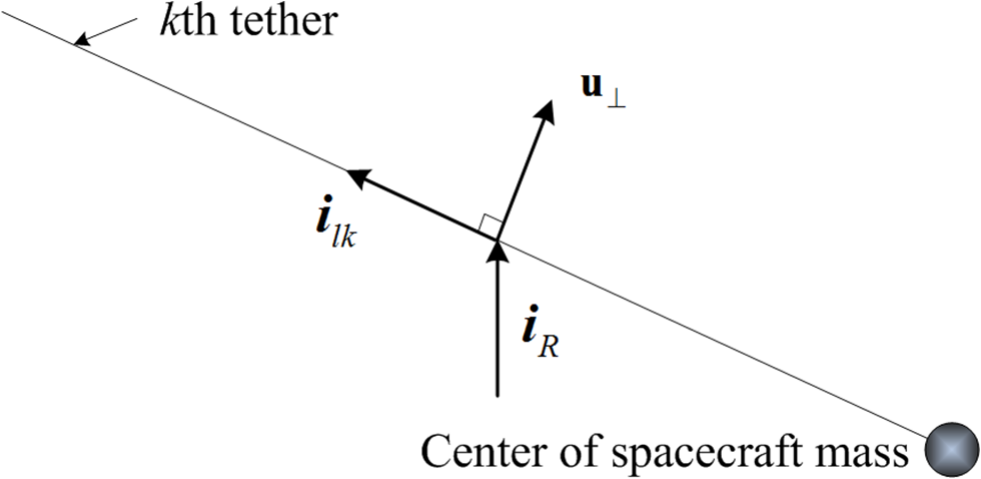
Thrust vector of *k*th tether.

The propulsive thrust per unit length of tether is given in [9] as

$$\frac{\text{d}F}{\text{d}l}=0.18\;\text{max}(0,V_{0}-V_{1})\sqrt{\varepsilon_{0}m_{p}n_{w}}u_{⊥}$$ (2.)

where *V*_0_ is the tether voltage, and *V*_1_ is the electric potential corresponding to the kinetic energy of the solar wind ions, *n*_*w*_ is the solar wind number density, and **u**_⊥_ is the component of the solar wind velocity which is perpendicular to the charged tether. As seen in Fig 4, **u**_⊥_ can be written as

$$u_{⊥}=ui_{l}\times i_{R}\times i_{l}$$ (3.)

where *u* is the magnitude of the solar wind velocity, ***i***_*l*_ is the unit direction vector of tether. ***i***_*R*_ is the unit vector of sun-spacecraft direction, and can be written as [0 0 1]^T^ in the orbital frame.

Because of the existence of centrifugally stabilizing auxiliary tethers [11], it can be assumed that electric sail tethers always distributed evenly in the *o*_*b*_ *x*_*b*_ *y*_*b*_ plane. Therefore, the unit direction vector of the *k*th tether in the body frame is [cos(2π(*k*-1)/*N*) sin(2π(*k*-1)/*N*) 0]^T^. Consequently, the unit direction vector of the *k*th tether in the orbital frame can be given by

$$i_{lk}=A_{ob}\left[\begin{array}{c} \text{cos}(2\pi (k-1)/N)\\ \text{sin}(2\pi (k-1)/N)\\ 0 \end{array}\right]$$ (4.)

Substituting Eq (3) into Eq (2), the thrust vector per unit length can be obtained as

$$\frac{\text{d}F}{\text{d}l}=\sigma i_{l}\times i_{R}\times i_{l}$$ (5.)

where $\sigma =0.18\;\text{max}(0,V_{0}-V_{1})\sqrt{\varepsilon_{0}m_{p}n_{w}u^{2}}$, is the magnitude of the thrust per unit length when the solar wind direction is perpendicular to the tether. Since the magnitude of the thrust per unit length is inversely proportional to the sun-sail distance *r* [6], the thrust vector per unit length of tether can be written as

$$\frac{\text{d}F}{\text{d}l}=\sigma_{⊕}\frac{r_{⊕}}{r}i_{l}\times i_{R}\times i_{l}$$ (6.)

where *σ*_⊕_ is the magnitude of the force per unit length, when the sun-spacecraft distance is *r*_⊕_ = 1au and the solar wind is perpendicular to tether. Note that *σ*_⊕_ can be controlled by altering the voltage of tethers.

Integrating Eq (6), the thrust vector of the *k*th tether can be obtained as

$$F_{k}=l\sigma_{⊕k}\left(\frac{r_{⊕}}{r}\right)i_{l}\times i_{R}\times i_{l}$$ (7.)

Summing over the thrust vector of single tethers, we can obtain the total thrust of the electric sail:

$$F(\phi ,\theta ,\psi ,r,l,\sigma_{⊕1},\ldots ,\sigma_{⊕N})=\sum_{k=1}^{N}F_{k}(\phi ,\theta ,\psi ,r,l,\sigma_{⊕k})$$ (8.)

If the voltage of each tether is the same, the vertical thrust magnitude of each tether will be *σ*_⊕1_ =…= *σ*_⊕*N*_ = *σ*_⊕_. When *N* is a multiple of 4, the thrust vector can be written in a quite simple form through the definite summation of Eq (8)

$$F=\frac{1}{2}Nl\sigma_{⊕}\left(\frac{r_{⊕}}{r}\right)\left[\begin{array}{c} \text{cos}\phi \text{sin}\theta \text{cos}\theta \\ -\text{sin}\phi \text{cos}\phi {\text{cos}}^{2}\theta \\ {\text{cos}}^{2}\phi {\text{cos}}^{2}\theta +1 \end{array}\right]$$ (9.)

As seen in Eq (9), when the voltage of each tether is the same, the thrust vector depends on the angular *ϕ* and *θ*, but not on *ψ*. It means that the rotation with respect to the normal of the sail (*z*_*b*_-axis) has no influence on the thrust vector.

Note that cos *α* = cos *ϕ*cos *θ*, where the angle *α* is the pitch angle between the sunlight and sail normal. Taking the scalar of Eq (9), the magnitude of thrust can be written as

$$F=\frac{1}{2}Nl\sigma_{⊕}\left(\frac{r_{⊕}}{r}\right)\sqrt{3{\text{cos}}^{2}\;\alpha +1}$$ (10.)

As seen in Eq (10), the thrust magnitude of electric sail depends on the pitch angle between the sunlight and sail normal. This phenomenon is the same as solar sails.

### Torque Vector

Charged tethers are not only able to generate the thrust, but also able to generate the torque [6]. Since the thrust magnitude depends on the tether potential, a potentiometer, placed between the spacecraft and each tether, can be used to generate control torque. Therefore, the sail plane attitude can be varied to turn the thrust direction under the control of torque [7].

The torque vector is perpendicular to the thrust vector. Considering Eq (6), the torque vector per unit tether length of the *k*th tether can be written as

$$\frac{\text{d}T_{k}}{\text{d}l}=li_{lk}\times \frac{\text{d}F_{k}}{\text{d}l}=l\sigma_{⊕k}\left(\frac{r_{⊕}}{r}\right)i_{lk}\times i_{R}$$ (11.)

Integrating Eq (11), the torque vector of the *k*th tether can be obtained as

$$T_{k}=\frac{1}{2}l^{2}\sigma_{⊕k}\left(\frac{r_{⊕}}{r}\right)i_{lk}\times i_{R}$$ (12.)

Summing over the torque vector of single tethers, the total torque of the electric sail can be written as

$$T(\phi ,\theta ,\psi ,r,l,\sigma_{⊕1},\ldots ,\sigma_{⊕N})=\sum_{k=1}^{N}T_{k}(\phi ,\theta ,\psi ,r,l,\sigma_{⊕k})$$ (13.)

Since all tethers’ torque vectors are perpendicular to the sun-sail direction (*z*_*o*_-axis) as seen in Eq (12), the component of torque vector of electric sail on the *z*_*o*_-axis is zero. Therefore, the torque vector in body frame can be written as

$${\left[\begin{array}{ccc} T_{bx} & T_{by} & T_{bz} \end{array}\right]}^{T}=A_{bo}{\left[\begin{array}{ccc} T_{ox} & T_{oy} & 0 \end{array}\right]}^{T}$$ (14.)

Then, the component of torque vector of electric sail on the *z*_*b*_ *-*axis are given by

$$T_{bz}=\frac{\text{tan}\;\phi \text{sin}\;\psi -\text{sin}\;\theta \text{cos}\;\psi }{\text{cos}\;\theta }T_{bx}+\frac{\text{tan}\;\phi \text{cos}\;\psi +\text{sin}\;\theta \text{sin}\;\psi }{\text{cos}\;\theta }T_{by}$$ (15.)

## Coupled Orbit–Attitude Dynamical Equations

As discussed in the previous section, the thrust vector of the electric sail depends on the sail attitude. Therefore, the orbital dynamics and attitude dynamics of electric sails are not completely independent. In this section, the coupled attitude-orbit dynamics is investigated.

### Orbital Dynamical Equations

In the investigation of orbital dynamics, the heliocentric inertial reference frame *o*_*i*_ *x*_*i*_ *y*_*i*_ *z*_*i*_ and the orbital rotating reference frame *o*_*o*_ *x*_*o*_ *y*_*o*_ *z*_*o*_ are used. As seen in Fig 3, the relative angular velocity between two reference frames can be given by

$$\omega_{o}={\left[\begin{array}{ccc} \dot{\Psi }\;\text{cos}\;\Theta & -\dot{\Psi }\;\text{sin}\;\Theta & \dot{\Theta } \end{array}\right]}^{\text{T}}$$ (16.)

where *Ψ* is the ecliptic longitude and *Θ* is the ecliptic latitude.

The vector equation of motion for the electric sail [10] is

$$\ddot{r}+2\omega_{o}\times \dot{r}+\omega_{o}\times (\omega_{o}\times r)=-\frac{\mu_{⊙}}{r^{3}}r+\frac{F}{m}$$ (17.)

where *m* is the total mass of the spacecraft, including the mass of spacecraft body, electric sail system and payload. *μ*_⊙_ is sun’s gravitational parameter.

Substituting Eqs (9) and (16) into Eq (17), the orbital dynamical scalar equations are

$$\{\begin{array}{l} \dot{r}=v_{r}\\ \dot{\Theta }=\omega_{\Theta }\\ \dot{\Psi }=\omega_{\Psi }\\ {\dot{v}}_{r}=r\omega_{\Psi }^{2}\;{\text{sin}}^{2}\;\Theta +r\omega_{\Theta }^{2}-\frac{\mu_{⊙}}{r^{2}}+\frac{\kappa a_{⊕}r_{⊕}}{2r}({\text{cos}}^{2}\;\phi {\text{cos}}^{2}\;\theta +1)\\ {\dot{\omega }}_{\Theta }=\omega_{\Psi }^{2}\;\text{sin}\;\Theta \text{cos}\;\Theta -\frac{2v_{r}\omega_{\Theta }}{r}+\frac{\kappa a_{⊕}r_{⊕}}{2r^{2}}(\text{cos}\;\phi \text{sin}\;\theta \text{cos}\;\theta )\\ {\dot{\omega }}_{\Psi }=-\frac{2v_{r}\omega_{\Psi }}{r}-2\omega_{\Theta }\omega_{\Psi }\text{cot}\;\Theta -\frac{\kappa a_{⊕}r_{⊕}}{2r^{2}}(\text{sin}\;\phi \text{cos}\;\phi {\text{cos}}^{2}\;\theta ) \end{array}$$ (18.)

where *a*_⊕_ = *Nl*σ_⊕_ / *m* is the characteristic acceleration of the electric sail, and can be controlled by altering the voltage of tethers. Since the thrust acceleration is induced by the attitude, the attitude will affect the orbit. This is also a common feature of all kinds of sail-spacecraft [10]. Thrust control coefficient *κ* ∈ [0, 1], as the thrust of the electric sail can be adjusted by the electron gun.

### Attitude Dynamical Equations

In the investigation of attitude dynamics, the orbital reference frame *o*_*o*_ *x*_*o*_ *y*_*o*_ *z*_*o*_ and the body reference frame *o*_*b*_ *x*_*b*_ *y*_*b*_ *z*_*b*_ are used. Based on the basic knowledge of attitude kinematics, the angular velocity of electric sail in the body reference frame can be written as

$$\left[\begin{array}{c} \omega_{bx}\\ \omega_{by}\\ \omega_{bz} \end{array}\right]=\left[\begin{array}{ccc} \text{cos}\;\theta \text{cos}\;\psi & \text{sin}\;\psi & 0\\ -\text{cos}\;\theta \text{sin}\;\psi & \text{cos}\;\psi & 0\\ \text{sin}\;\theta & 0 & 1 \end{array}\right]\left[\begin{array}{c} \dot{\phi }\\ \dot{\theta }\\ \dot{\psi } \end{array}\right]+A_{{}_{bo}}\left[\begin{array}{c} -\omega_{\Psi }\;\text{sin}\;\Theta \\ \omega_{\Theta }\\ \omega_{\Psi }\;\text{cos}\;\Theta \end{array}\right]$$ (19.)

Consider Eq (19), the kinematical differential equation can be obtained as

$$\left[\begin{array}{c} \dot{\phi }\\ \dot{\theta }\\ \dot{\psi } \end{array}\right]=\frac{1}{\text{cos}\;\theta }\left[\begin{array}{ccc} \text{cos}\;\psi & -\text{sin}\;\psi & 0\\ \text{cos}\;\theta \text{sin}\;\psi & \text{cos}\;\theta \text{cos}\;\psi & 0\\ -\text{sin}\;\theta \text{cos}\;\psi & \text{sin}\;\theta \text{sin}\;\psi & \text{cos}\;\theta \end{array}\right]\left[\begin{array}{c} \omega_{bx}\\ \omega_{by}\\ \omega_{bz} \end{array}\right]+\frac{1}{\text{cos}\;\theta }\left[\begin{array}{ccc} -\text{cos}\;\theta & -\text{sin}\;\phi \text{sin}\;\theta & \text{cos}\;\phi \text{sin}\;\theta \\ 0 & -\text{cos}\;\phi \text{cos}\;\theta & -\text{sin}\;\phi \text{cos}\;\theta \\ 0 & \text{sin}\;\phi & -\text{cos}\;\phi \end{array}\right]\left[\begin{array}{c} -\omega_{\Psi }\;\text{sin}\;\Theta \\ \omega_{\Theta }\\ \omega_{\Psi }\;\text{cos}\;\Theta \end{array}\right]$$ (20.)

Based on the basic knowledge of attitude dynamics, the attitude equations of motion in body frame are given by

$$I{\dot{\omega }}_{b}+\omega_{b}\times (I\omega_{b})=T$$ (21.)

where ***I*** is the inertia matrix of spacecraft. Assuming the inertia matrix ***I*** is a diagonal matrix, the angular acceleration expressed in body frame can be obtained as

$$\left[\begin{array}{c} {\dot{\omega }}_{bx}\\ {\dot{\omega }}_{by}\\ {\dot{\omega }}_{bz} \end{array}\right]=\left[\begin{array}{ccc} 1/I_{x} & 0 & 0\\ 0 & 1/I_{y} & 0\\ 0 & 0 & 1/I_{z} \end{array}\right]\left[\begin{array}{c} T_{x}\\ T_{y}\\ T_{z} \end{array}\right]+\left[\begin{array}{c} \omega_{by}\omega_{bz}(I_{y}-I_{z})/I_{x}\\ \omega_{bx}\omega_{bz}(I_{z}-I_{x})/I_{y}\\ \omega_{bx}\omega_{by}(I_{x}-I_{y})/I_{z} \end{array}\right]$$ (22.)

Substitution of Eqs (19) and (22) into the attitude dynamical Eq (21) generates the attitude dynamical equations as

$$\{\begin{array}{l} \;\dot{\phi }=\omega_{bx}\;\text{cos}\;\psi /\text{cos}\;\theta -\omega_{by}\;\text{sin}\;\psi /\text{cos}\;\theta +\omega_{\Psi }\;\text{sin}\;\Theta -\omega_{\Theta }\;\text{sin}\;\phi \text{tan}\;\theta +\omega_{\Psi }\;\text{cos}\;\Theta \;\text{cos}\;\phi \text{tan}\;\theta \\ \;\dot{\theta }=\omega_{bx}\;\text{sin}\;\psi +\omega_{by}\;\text{cos}\;\psi -\omega_{\Theta }\;\text{cos}\;\phi -\omega_{\Psi }\;\text{cos}\;\Theta \text{sin}\;\phi \\ \;\dot{\psi }=-\omega_{bx}\;\text{tan}\;\theta \text{cos}\;\psi +\omega_{by}\;\text{tan}\;\theta \text{sin}\;\psi +\omega_{bz}+\omega_{\Theta }\text{sin}\;\phi /\text{cos}\;\theta -\omega_{\Psi }\;\text{cos}\;\Theta \text{cos}\;\phi /\text{cos}\;\theta \\ {\dot{\omega }}_{bx}=\omega_{by}\omega_{bz}\left(I_{y}-I_{z}\right)/I_{x}+T_{x}/I_{x}\\ {\dot{\omega }}_{by}=\omega_{bx}\omega_{bz}\left(I_{z}-I_{x}\right)/I_{y}+T_{y}/I_{y}\\ {\dot{\omega }}_{bz}=\omega_{bx}\omega_{by}\left(I_{x}-I_{y}\right)/I_{z}+\left(\left(\text{tan}\;\phi \text{sin}\;\psi -\text{sin}\;\theta \text{cos}\;\psi \right)T_{x}+\left(\text{tan}\;\phi \text{cos}\;\psi +\text{sin}\;\theta \text{sin}\;\psi \right)T_{y}\right)/\left(\text{cos}\;\theta I_{z}\right) \end{array}$$ (23.)

## Orbit and Attitude Control

**Fig 5:**
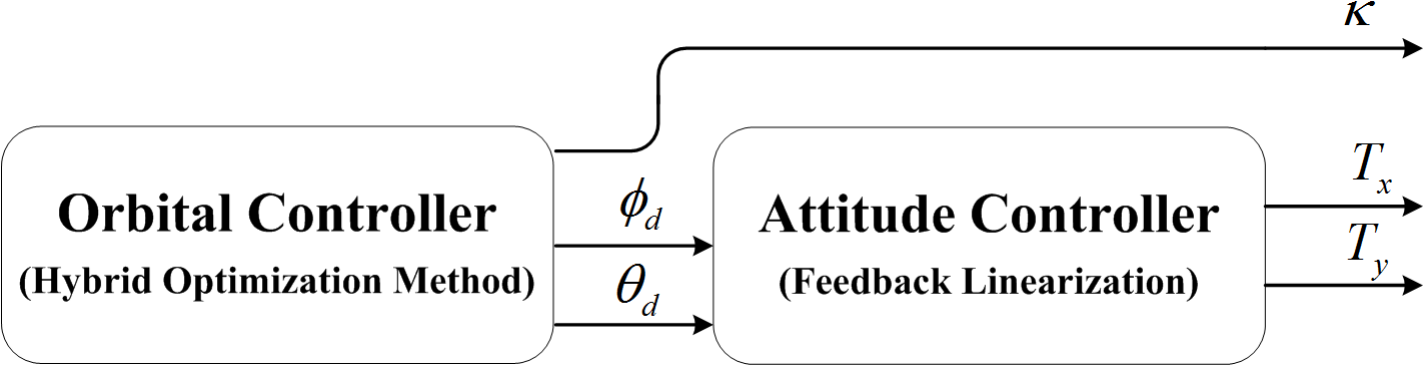
The flow chat of orbital and attitude control.

Based on the coupled equations, the orbit and attitude control for the minimum-time electric sail transfer problem from Earth’s orbit to a target planet are investigated in this section. As seen in Fig 5, the orbital control is studied in an optimal framework via a hybrid optimization method and the attitude controller is designed based on feedback linearization control. The main idea of orbital and attitude control for electric sail is shown in Fig 5, and the detailed implementation can be seen in S1 File.

### Orbital Control via a Hybrid Optimization Method

Since the orbit is determined by the attitude history, the orbital control is studied in an optimal framework via a hybrid optimization method to generate the optimal attitude. In the orbital control of electric sail, the state variables are orbital parameters [*r*, *Θ*, *Ψ*, *v*_*r*_, *ω*_*Θ*_, *ω*_*Ψ*_]^T^. The control variables are thrust control coefficient *κ* and attitude angles *ϕ*, *θ*, which can influence the thrust vector. In the trajectory optimization for spacecraft, the objective function to be minimized usually is fuel consumption or transfer time. Since the electric sail can use the solar-wind-dynamic pressure to generate thrust without fuel consumption, the transfer time is always selected as the optimization performance index to be minimized [1], [7].

$$J=t_{f}-t_{0}$$ (24.)

where *t*_*f*_ is the final time and *t*_0_ is the initial time. The *t*_0_ is a variable needs to be optimized in the process to obtain the optimal launch window.

Assuming that the electric sail is in the Earth's orbit at the initial time *t*_0_, the initial state of the electric sail should be consistent with the orbit parameters of Earth and can be written as

$$\{\begin{array}{ccc} r(t_{0})=r_{⊕}(t_{0}) & \Theta (t_{0})=\Theta_{⊕}(t_{0}) & \Psi (t_{0})=\Psi_{⊕}(t_{0})\\ v_{r}(t_{0})=v_{r}{}_{⊕}(t_{0}) & \omega_{\Theta }(t_{0})=\omega_{\Theta }{}_{⊕}(t_{0}) & \omega_{\Psi }(t_{0})=\omega_{\Psi }{}_{⊕}(t_{0}) \end{array}$$ (25.)

where *r*_⊕_(*t*_0_), *Θ*_⊕_(*t*_0_), *Ψ*_⊕_(*t*_0_), *v*_*r*⊕_(*t*_0_), *ω*_Θ⊕_(*t*_0_) and *ω*_Ψ⊕_(*t*_0_) are the orbital parameters of Earth.

Assuming the electric sail arrived in the orbit of target planet at the final time *t*_*f*_, the final state of the electric sail should be consistent with the orbit parameters of target planet and can be written as

$$\{\begin{array}{ccc} r(t_{f})=r_{⊗}(t_{f}) & \Theta (t_{f})=\Theta_{⊗}(t_{f}) & \Psi (t_{f})=\Psi_{⊗}(t_{f})\\ v_{r}(t_{f})=v_{r}{}_{⊗}(t_{f}) & \omega_{\Theta }(t_{f})=\omega_{\Theta }{}_{⊗}(t_{f}) & \omega_{\Psi }(t_{f})=\omega_{\Psi }{}_{⊗}(t_{f}) \end{array}$$ (26.)

where *r*_⊗_(*t*_*f*_), *Θ*_⊗_(*t*_*f*_), *Ψ*_⊗_(*t*_*f*_), *v*_*r*⊗_(*t*_*f*_), *ω*_Θ⊗_(*t*_*f*_) and *ω*_Ψ⊗_(*t*_*f*_) are the orbital parameters of the target planet which can be calculated from orbital elements.

**Fig 6:**
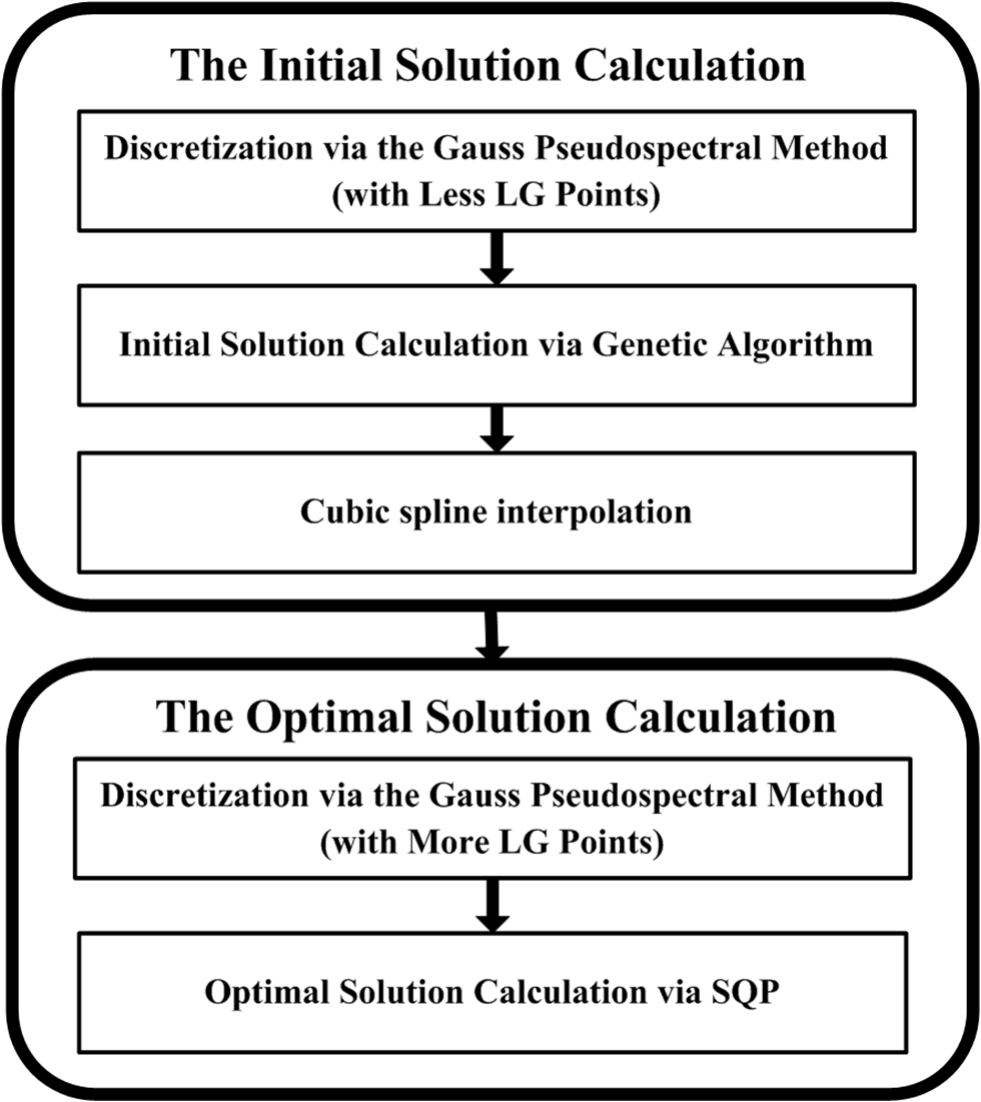
The flow chart of hybrid optimization method.

For the orbital control problem of electric sails, the hybrid genetic algorithm Gauss pseudospectral optimization method is implemented in this paper. As seen in Fig 6, the initial guesses for the state and control histories of the nonlinear programming problem (NLP), which is transcribed from the continuous optimal control problem by using Gauss pseudospectral method [12], are interpolated from the best solution of a genetic algorithm (GA) with less Legendre-Gauss (LG) points. Through a random and global search, GA can provide a reasonable initial guess. Based on the obtained initial guess, the NLP can be solved by sequential quadratic programming (SQP) to obtain the optimal solution. Therefore, the hybrid optimization method is capable of searching the feasible and optimal solution without any initial value guess. This feature is ideal for the orbital control problem of electric sails, which is generally without prior knowledge. The indirect optimization method is popular method used in previous researches on the orbital control of electric sail. However, it is sensitive to the initial value of co-state variable. Compared with it, this hybrid optimization method overcomes the shortcomings. Moreover, compared with the regular Gauss pseudospectral method, the hybrid optimization method does not need any initial guess to search the feasible and optimal solution. This feature is very suitable for trajectory optimization problems of electric sail, which are short of priori knowledge. Besides, the hybrid optimization method also has the capability to global search, instead of local search in the regular Gauss pseudospectral method. The hybrid optimization method is summarized in this section, and a thorough discussion can be found in [13].

### Attitude Control via Feedback Linearization

As seen in attitude dynamical equations Eq (23), there is strong nonlinearity and coupling while the attitude angle of sailcraft is large. Therefore, the traditional approximate linearization method based on small disturbance faces difficulties. To track the referenced attitude trajectory, the attitude controller is designed based on feedback linearization control (FBL). General FBL is well suited for complex non-square nonlinear models, making time-scale separation unnecessary. Using Lie algebra, one nonlinear state feedback control law is derived which cancels the nonlinearities of both the rotational dynamics and kinematics [14]. In this section, the FBL theory is summarized. A thorough discussion can be found in [15].

As described in Eq (23), the state vector ***x*** can be defined as ***x*** = [*ϕ θ ψ ω*_*bx*_ *ω*_*by*_ *ω*_*bz*_ ]^*T*^. Since the attitude angel *ϕ* and *θ* can influence the orbit, the output vector ***y*** is defined as ***y*** = [*ϕ θ*]^*T*^ and the input vector ***u*** is defined as ***u*** = [*T*_*x*_ *T*_*y*_]^*T*^. Considering the attitude dynamical equations Eq (23), the attitude control problem can be written as

$$\{\begin{array}{l} \dot{x}=f(x)+g(x)u\\ y=h(x) \end{array}$$ (27.)

where \

$$\begin{array}{c} f(x)=\left[\begin{array}{c} x_{4}\text{cos}\;x_{3}/\text{cos}\;x_{2}-x_{5}\text{sin}\;x_{3}/\text{cos}\;x_{2}+\omega_{\Psi }\text{sin}\;\Theta -\omega_{\Theta }\text{sin}\;x_{1}\text{tan}\;x_{2}+\omega_{\Psi }\text{cos}\;\Theta \text{cos}\;x_{1}\text{tan}\;x_{2}\\ x_{4}\text{sin}\;x_{3}+x_{5}\text{cos}\;x_{3}-\omega_{\Theta }\text{cos}\;x_{1}-\omega_{\Psi }\text{cos}\;\Theta \text{sin}\;x_{1}\\ -x_{4}\text{tan}\;x_{2}\text{cos}\;x_{3}+x_{5}\text{tan}\;x_{2}\text{sin}\;x_{3}+x_{6}+\omega_{\Theta }\text{sin}\;x_{1}/\text{cos}\;x_{2}-\omega_{\Psi }\text{cos}\;\Theta \text{cos}\;x_{1}/\text{cos}\;x_{2}\\ x_{5}x_{6}\left(I_{y}-I_{z}\right)/I_{x}\\ x_{4}x_{6}\left(I_{z}-I_{x}\right)/I_{y}\\ x_{4}x_{5}\left(I_{x}-I_{y}\right)/I_{z} \end{array}\right]\\ g(x)={\left[\begin{array}{cccccc} 0 & 0 & 0 & 1/I_{x} & 0 & \left(\text{tan}\;x_{1}\text{sin}\;x_{3}-\text{sin}\;x_{2}\text{cos}\;x_{3}\right)/\left(\text{cos}\;x_{2}I_{z}\right)\\ 0 & 0 & 0 & 0 & 1/I_{y} & \left(\text{tan}\;x_{1}\text{cos}\;x_{3}+\text{sin}\;x_{2}\text{sin}\;x_{3}\right)/\left(\text{cos}\;x_{2}I_{z}\right) \end{array}\right]}^{\text{T}},h(x)=\left[\begin{array}{c} x_{1}\\ x_{2} \end{array}\right] \end{array}$$

It has been obtained that nonlinear system has the vector relative degree {2 2}. Therefore, the feedback functions Λ(***x***) and ***B***(***x***) are defined as following,

$$\begin{array}{cc} \Lambda (x)=\left[\begin{array}{cc} L_{g1}L_{f}h_{1}(x) & L_{g2}L_{f}h_{1}(x)\\ L_{g1}L_{f}h_{2}(x) & L_{g2}L_{f}h_{2}(x) \end{array}\right] & \;B(x)=\left[\begin{array}{c} L_{f}^{2}h_{1}(x)\\ L_{f}^{2}h_{2}(x) \end{array}\right] \end{array}$$ (28.)

where *L*_*gi*_ *L*_*f*_ *h*_*j*_ (***x***) and $L_{f}^{2}h_{j}(x)$ are Lie derivatives of the scalar functions *h*_*j*_(***x***) with respect to the vectors ***f***(***x***) and ***g***_*i*_(***x***), with *j*, *i* = 1 to 2. When *θ* is not equal to *π /* 2, the matrix Λ(***x***) is nonsingular. The state feedback can be applied to the nonlinear system,

$$u=\Lambda {\left(x\right)}^{-1}(\upsilon -B(x))$$ (29.)

The nonlinear system (26) could be transformed to a linear one in the form of,

$${\left[\begin{array}{cc} {\ddot{y}}_{1} & {\ddot{y}}_{2} \end{array}\right]}^{T}={\left[\begin{array}{cc} \upsilon_{1} & \upsilon_{2} \end{array}\right]}^{T}$$ (30.)

The system now consists of two second-order decomposed channels which can be controlled by two independent controllers. Based on the pole assignment method, the new control variables can be selected as

$$\left[\begin{array}{c} \upsilon_{1}\\ \upsilon_{2} \end{array}\right]=\left[\begin{array}{c} {\ddot{\varphi }}_{d}+c_{1}({\dot{\phi }}_{d}-{\dot{y}}_{1})+c_{2}(\phi_{d}-y_{1})\\ {\ddot{\theta }}_{d}+c_{3}({\dot{\theta }}_{d}-{\dot{y}}_{2})+c_{4}(\theta_{d}-y_{2}) \end{array}\right]$$ (31.)

where *ϕ*_*d*_ and *θ*_*d*_ are the referenced attitude angles generated by orbital controller, and *c*_1_, *c*_2_, *c*_3_, *c*_4_ > 0 is attitude controller parameters.

## Numerical Simulations

To verify the effectiveness of the proposed control strategy, a transfer problem from Earth to Mars is investigated. The simulation parameters of the electric sail are shown in the Table 1. The characteristic acceleration *a*_⊕_ = 2mm/s^2^ corresponds to an estimated maximum value of the propelling acceleration at *r* = *r*_⊕_ that will probably be available in a near future [7]. In orbital control, the histories of referenced attitude angles *ϕ*_*d*_, *θ*_*d*_ and proportionality coefficient *κ* are generated by the hybrid optimization method. In the initial solution calculation via GA, the quantity of LG points is 30. The population size is 100, and the number of generations is 200. In the optimal solution calculation via SQP, the quantity of LG points is 90. The convergence criteria for the NLP are a maximum constraint violation of 1×10^−9^ (TolCon) and a reduction in the cost function of less than 1×10^−6^ (TolFun) for one iteration. In attitude control, controller parameters are selected as *c*_1_, *c*_3_ = 5×10^−5^ and *c*_2_, *c*_4_ = 2.5×10^−10^. The initial attitude angles are assumed to be equal to the referenced attitude angles. The detailed implementation can be seen in the simulation program (S1 File).

**Table 1:** Simulation parameters of the electric sail.

Symbol	Value
*a*_⊕_	2mm/s^2^
*I*_*x*_, *I*_*y*_	7.333×10^8^kgm^2^
*I*_*z*_	14.667×10^8^kgm^2^
*N*	100
*l*	10km

**Fig 7:**
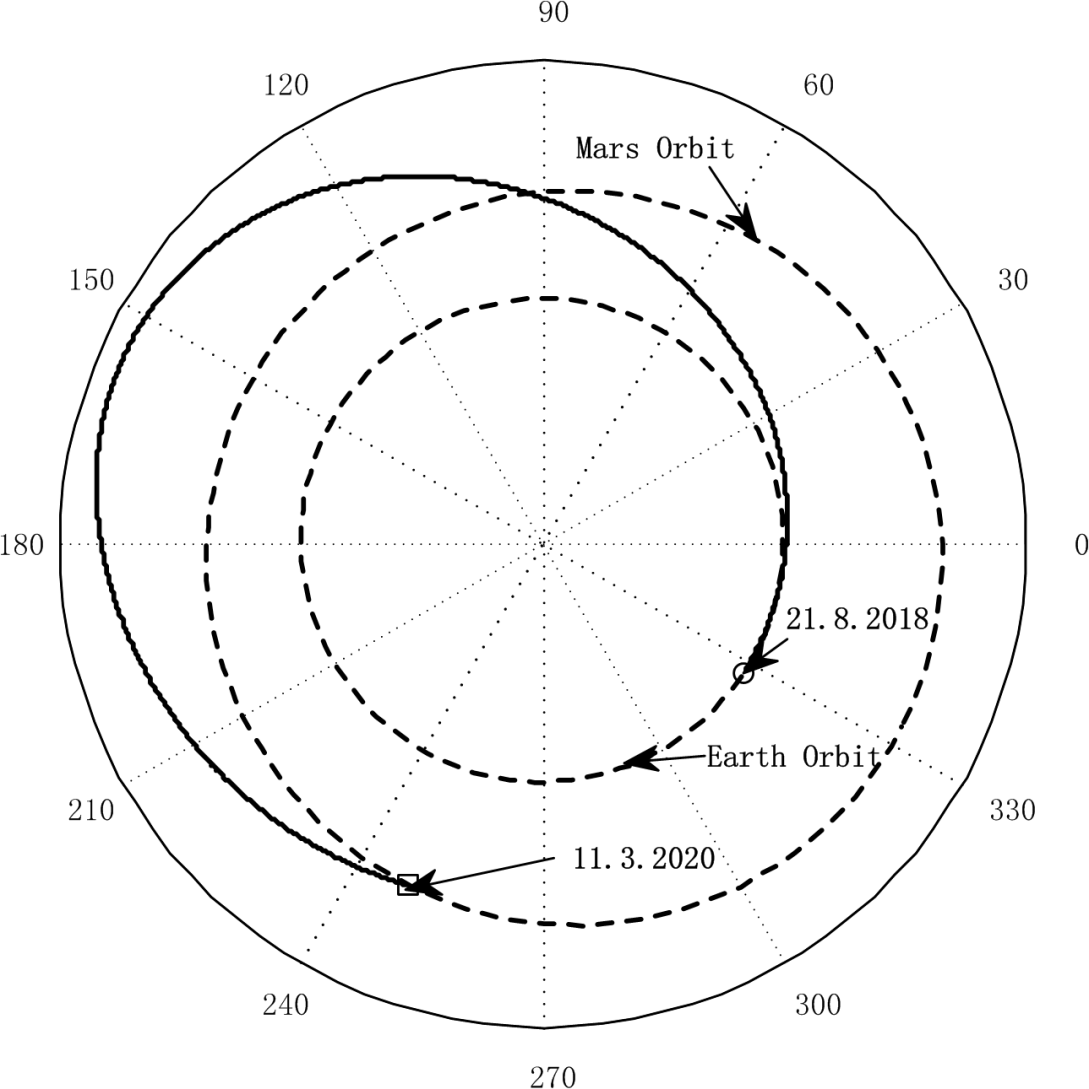
Earth-Mars transfer trajectory.

**Fig 8:**
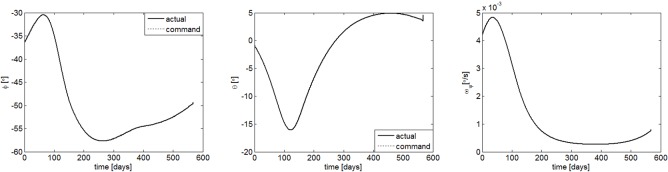
Attitude responses and spin velocity.

**Fig 9:**
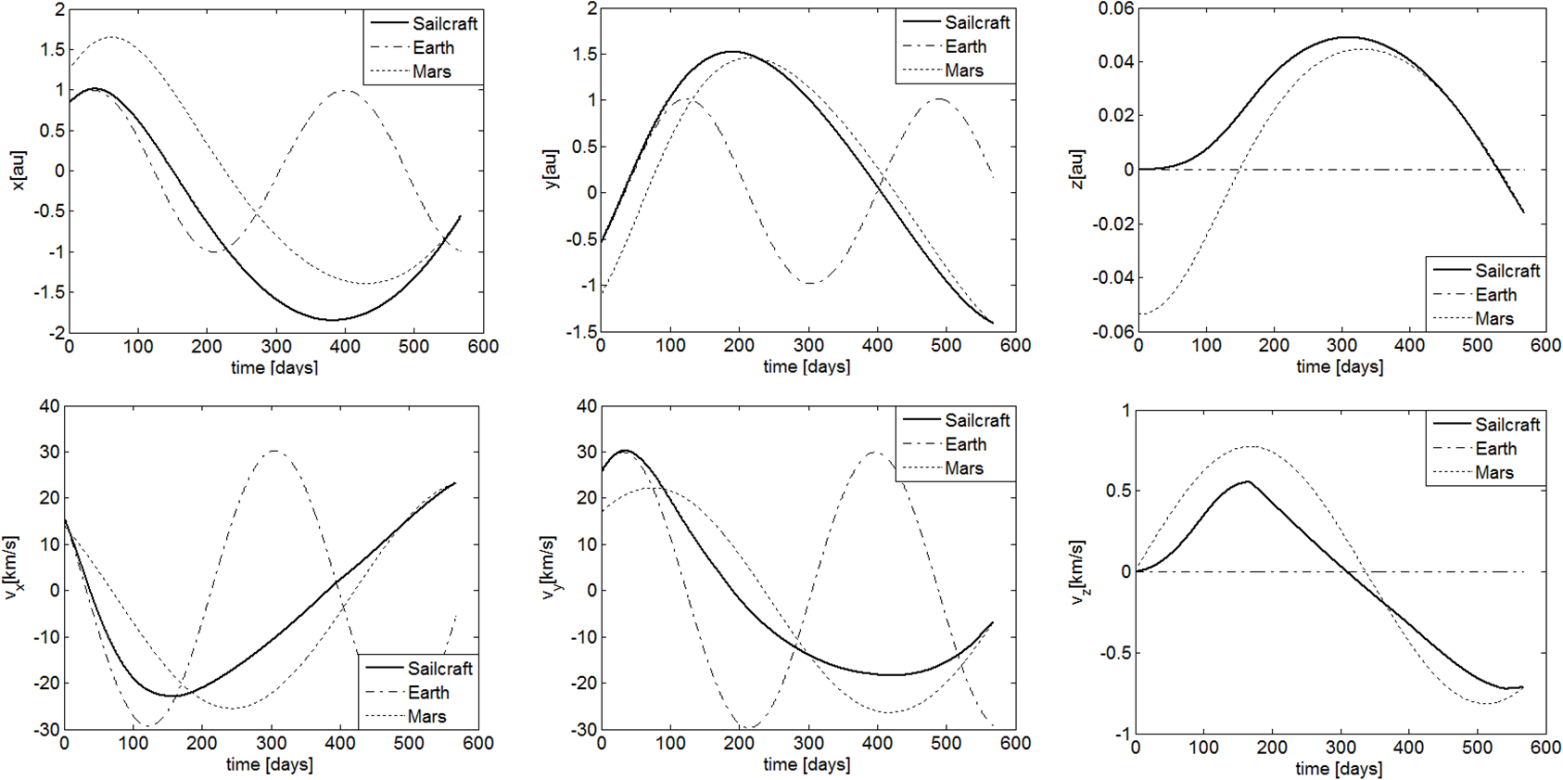
Position and velocity histories in the inertial frame *o*_*i*_ *x*_*i*_ *y*_*i*_ *z*_*i*_.

**Fig 10:**
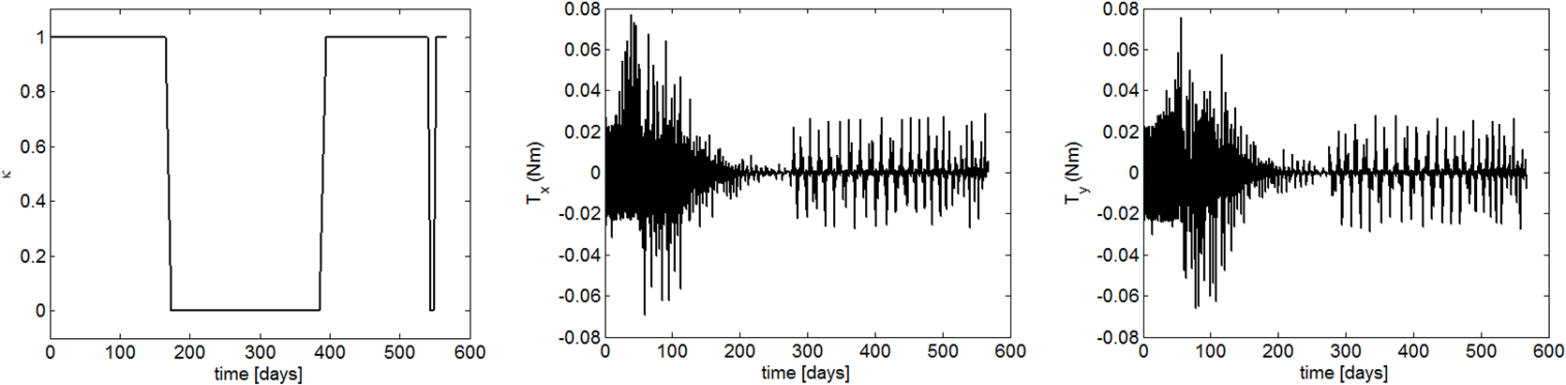
Control histories.

The transfer trajectory from Earth to Mars is shown in Fig 7. If the sailcraft left the sphere of influence of Earth at August 21, 2018, it will take 567.6 days to get into the sphere of influence of Mars. The departure time is a launch window which is obtained through the hybrid optimization method. Fig 8 gives the commanded and the response of the controlled attitude. It can be seen that the command attitude angle histories, generated by the hybrid optimization method, is smooth and continuous. Furthermore, the sail attitude follows the commanded attitude closely under the FBL control law, and the spinning angular velocity fluctuated within an acceptable range. Fig 9 gives the response of the orbit. They are shown that the position and velocity meet the boundary constrains very well. This means that the sailcraft successfully entered the sphere of influence for Mars with low relative velocity. As seen in Fig 10, the maximum control torque is about 0.076Nm, which is quite small for electric sail spacecraft. Overall, the numerical results demonstrate that the proposed strategy is capable of controlling both the attitude and orbit of the coupled system with only a small control torque.

## Conclusions

The results of this paper are based on a simple dynamical model for the electric sail tether rotating under constant solar wind forcing. In the paper, it is assumed that the structure of the sail is invariant and well tightened by the centrifugal force and auxiliary tethers. For electric sails, the orbit and attitude are not completely independent on each other, since the solar-wind-dynamic pressure acceleration induced by the sail attitude will decide the orbit. Therefore, the orbital dynamics and attitude dynamics are discussed together. Based on the coupled equations, the flight control is investigated, wherein the orbital control is studied in an optimal framework via a direct optimization method and the attitude controller is designed based on feedback linearization control. To verify the effectiveness of control, a transfer problem from Earth to Mars is considered. The numerical result shows that the proposed controllers can control the coupled system very well, and a small control torque can control both the attitude and orbit. Although it is still deserved more refined theoretical and experimental studies for the electric sail, the proposed research represents a first step for the preliminary flight control of this innovative propulsion concept.

## Supporting Information

S1 File The simulation program. (ZIP)
